# Analysing the integrated quorum sensing (iqs) system and its potential role in *Pseudomonas aeruginosa* pathogenesis

**DOI:** 10.3389/fcimb.2025.1575421

**Published:** 2025-05-14

**Authors:** Juan Raya, Enrique-J. Montagut, M.-Pilar Marco

**Affiliations:** ^1^ Department of Chemical and Biomolecular Nanotechnology, Nanobiotechnology for Diagnostics (Nb4D), Institute for Advanced Chemistry of Catalonia (IQAC) of the Spanish Council for Scientific Research (CSIC), Barcelona, Spain; ^2^ CIBER de Bioingeniería, Biomateriales y Nanomedicina (CIBER-BBN), Barcelona, Spain

**Keywords:** *P. aeruginosa*, quorum sensing, IQS, autoinducers, aeruginaldehyde

## Abstract

Since its discovery, Quorum Sensing (QS), a form of bacterial communication, has been the focus of numerous studies aimed at unravelling the mechanisms behind this intricate process. Bacterial QS relies on releasing low molecular weight signals known as autoinducers (AIs). When these AIs reach a threshold concentration, they activate coordinated genetic expression of pathogenic and bacterial survival mechanisms. *Pseudomonas aeruginosa*’s QS has been extensively studied due to its incidence and clinical significance in a wide range of human infections. Several decades ago, three QS systems, named Las, Rhl, and Pqs, were identified and have since then become the focus of numerous research studies and the target of innovative diagnostic and therapeutic strategies. However, a fourth QS-related system was more recently proposed that it has been the subject of debate. Named “*integrated quorum sensing*” (Iqs), interconnects the previously mentioned systems with the phosphate stress response. The associated AI has been identified as 2-(2-hydroxyphenyl)-thiazole-4-carbaldehyde, also known as IQS. This discovery has sparked a controversial discussion about its biosynthetic origin and whether it truly functions as an intercellular communication system. In this review, we critically discuss the different hypotheses, and its biological relevance while presenting key findings of the Iqs system.

## 1 Introduction

*P. aeruginosa* is a Gram-negative bacterium capable of causing a wide array of infections due to its unique ability to adapt and thrive in hostile environments. This ubiquitous and opportunistic pathogen often profits the breaches in the tissue barriers and overcomes the host immune system response through a diverse set of biological mechanisms. It especially affects individuals with a compromised immune system or other underlying health conditions, such as cystic fibrosis (CF) (Alhazmi, 2015). Furthermore, the isolated pathogenic strains are normally multi-drug resistant and lead to severe and persistent infections. Consequently, *P. aeruginosa* is one of the most commonly isolated microorganisms in healthcare-associated and community-acquired infections (Horcajada et al., 2019). The incidence and prevalence of this bacterium have prompted a global health and economic burden for which conventional therapeutic options are becoming limited (Judd et al., 2016).

*P. aeruginosa* also poses significant challenges in the food industry. It can contaminate food products, including drinking water, bottled water, and food processing areas, contributing to spoilage and potential foodborne and waterborne illnesses. It is frequently found in dairy products, meat, and seafood due to contamination from water sources or improper handling. Its biofilm-forming ability on food surfaces and processing equipment complicates eradication and heightens the risk of cross-contamination. *P. aeruginosa* is a common indicator of water quality issues and is often used as a marker for biofilm formation in drinking water systems. It can persist in treated water supplies, pools, and hot tubs, leading to skin infections, ear infections, and respiratory illnesses. Additionally, it raises concerns about the possibility of spreading antimicrobial resistance through the food chain. Managing its presence through proper water treatment, food hygiene, sanitation of food processing and water distribution equipment and suitable monitoring programs is crucial for protecting public health (Rather et al., 2021; Li et al., 2022).

One of the key contributors to the pathogenicity of *P. aeruginosa* is its extensive arsenal of virulence factors. These include extracellular enzymes (e.g., elastase, alkaline protease, phospholipase C), toxins (e.g., exotoxin A, pyocyanin), adhesins (e.g., type IV pili, flagella), and siderophores (pyoverdine and pyochelin) that aid in iron acquisition. Additionally, rhamnolipid production facilitates immune evasion and biofilm formation, a hallmark of *P. aeruginosa* infections (Qin et al., 2022).

Biofilms are structured microbial communities encased in an extracellular polymeric substance, enhancing antibiotic resistance and immune evasion. *P. aeruginosa* biofilms play a crucial role in chronic infections, particularly in CF patients’ lungs, burn wounds, and medical devices such as catheters and ventilators. The ability to form biofilms is tightly regulated by its Quorum Sensing (QS) system, which allows bacterial populations to coordinate gene expression based on cell density (Rather et al., 2021).Due to the clinical relevance of *P. aeruginosa*, its QS system has been widely studied. One of the reasons for the extraordinary effectiveness of *P. aeruginosa* pathogenicity is the QS, which allows the bacterium to communicate and adapt to different environments. This communication system in bacteria was discovered more than 40 years ago by Nealson and co-workers in the marine symbiotic bacterium *Vibrio fischeri* during the study of population density-dependent activation of bioluminescence (Nealson et al., 1970). This study showed that the bioluminescent process was only activated when the population density reached a certain level and there were enough bacteria to carry out the process efficiently. During the following years, it was demonstrated that the QS communication process enables collective activation and gene expression related to a large number of bacterial mechanisms. This process is based on the biosynthesis, release, and detection of low molecular weight signals called autoinducers (AIs) (Turan et al., 2017). These AI are released into the extracellular space and sensed by the surrounding microbial community. Then, when a certain threshold is reached, the coordinated genetic expression is triggered. In a similar manner to *Vibrio fischeri*, pathogenic bacteria drive group behaviour and collectively respond to host environmental stress through QS, or else the individual performance of such processes would be ineffective.

Due to the relevance of *P. aeruginosa* as a pathogenic microorganism, its QS system has been widely studied. It presents a particularly fine-tuned and convoluted network in which at least three interconnected systems (Las, Rhl and Pqs) perform in a hierarchical manner (Lee and Zhang, 2015). Each system produces and responds to a characteristic AI that enters into the cytoplasm and binds to its corresponding cognate transcriptional activator (LasR, RhlR, PqsR). Afterwards, upon binding of the complexes to the gene promoters, the expression of the associated regulons for their biosynthesis is triggered (LasI, RhlI, PqsABCD, respectively), generating an auto-induction loop that dramatically increases the number of QS signals. In addition to this, each transcriptional activator-AI complex controls the expression of a wide variety of genes related to virulence, biofilm formation, and secondary metabolism (Papenfort and Bassler, 2016).

**Figure 1:**
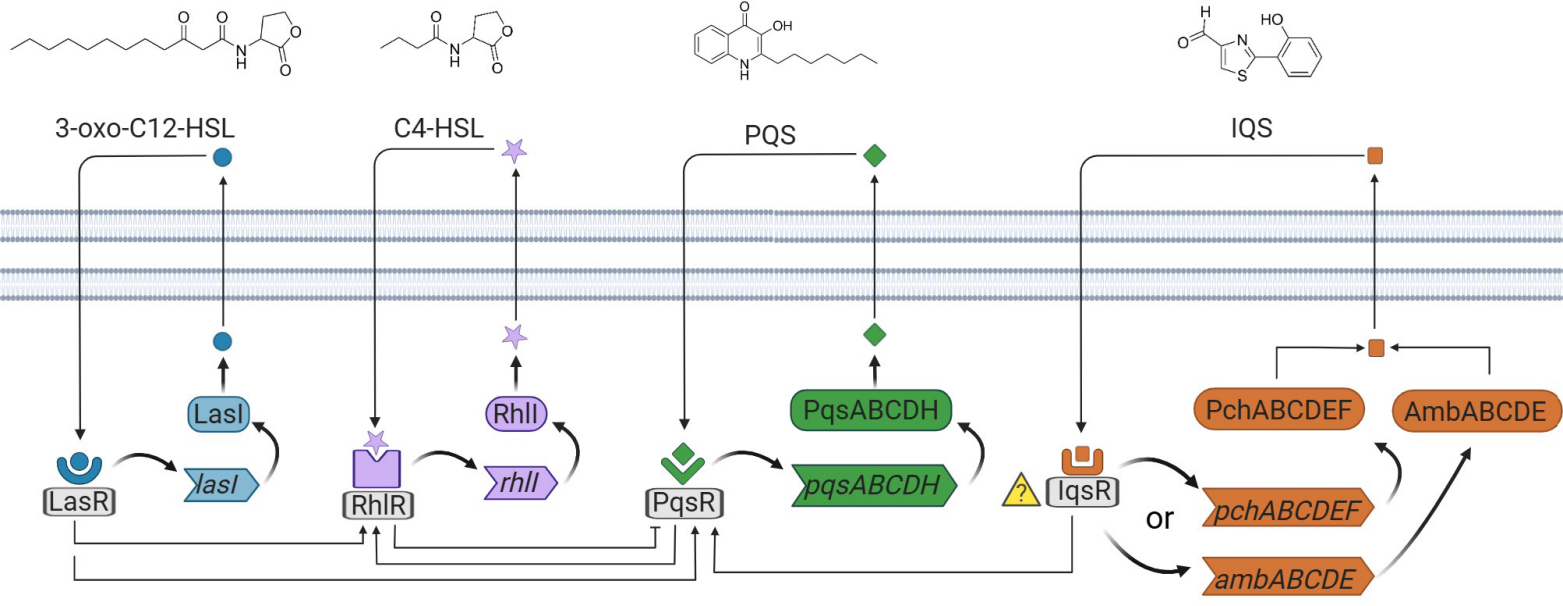
Schematic representation of the four QS networks in *P. aeruginosa*. The four autoinducer synthases produce the autoinducers, 3-oxo-C12-HSL, C4-HSL, PQS, and IQS. 3-oxo-C12-HSL, C4-HSL, and PQS are recognized by the receptors placed in the cytoplasm. The receptor for IQS is currently unknown.

The structure of the first chemical signal discovered in *P. aeruginosa* was elucidated more than 30 years ago by Pearson and co-workers, corresponding to N-(3-oxododecanoyl)-L-homoserine lactone (3-oxo-C12-HSL) (Pearson et al., 1994). This molecule belongs to the *las* signaling system, which indeed stands at the top of the regulation network, exerting positive control over the rest of the systems. The following system found to play a role in the *P. aeruginosa* QS was *rhl (* Ochsner and Reiser, 1995). It relies on a structurally related AI, N-butyryl-L-homoserine lactone (C4-HSL), discovered by Ochsner and co-workers. The complex C4-HSL -RhlR is responsible for exerting negative control over a third system, later named Pqs. The relevance and implication of the Pqs system and the role of 2-heptyl-3-hydroxy-4(1H)-quinolone or PQS (Pseudomonas quinolone signal), was enlightened by Pesci and co-workers in 1999 (Pesci et al., 1999). The complex autoinducer/transcriptional activator PQS-PqsR in turn activates the expression of RhlI, therefore increasing the overall expression of the *Rhl* system. This system controls a great number of QS-dependent virulence factors (See **Figure 1**).

The aforementioned systems were discovered within a relatively short timeframe; however, what was initially considered the fourth QS system was reported more than a decade later (Lee et al., 2013). The *integrated quorum sensing* (Iqs) system was initially described to connect the central Las system and phosphate stress response with the Pqs and Rhl downstream regulatory systems. The characteristic signaling molecule of the Iqs QS system corresponds to 2-(2-hydroxyphenyl)-thiazole-4-carbaldehyde or IQS. Interestingly, its structure and production by *P. aeruginosa* had been elucidated many years before uncovering its potential implication in the communication architecture (Yang et al., 1993). Previously, it was thought to be merely a degradation product from aeruginoic acid in the biosynthesis of the siderophore pyochelin (PCH). Indeed, the implicated genes in the biosynthesis and production of IQS have sparked debate regarding whether it follows the PCH-related production route or the ambBCDE cluster (Cornelis, 2020), which is thought to be responsible for the synthesis of the AMB antimetabolite. For that reason and due to the importance of Iqs system on the regulation of the QS of *P. aeruginosa*, in this review we give a critical overview of the latter QS fundamental findings, including biosynthetic pathways hypothetically involved, the biological significance of the IQS signaling molecule and techniques developed for its detection.

## 2 IQS system discovery

*P. aeruginosa* increases in prevalence during the lifetime of CF patients and is associated with accelerated decline in lung function. Clinical isolates obtained from CF patients with loss of function mutations in the central Las regulatory system are frequently found. The presence of *lasR* mutants has been envisaged as an adaptation to specific environments, and several studies have linked these mutations to worsening disease progression in acute or chronic infections (Dekimpe and Deziel, 2009; Hoffman et al., 2009; Wang et al., 2018). Interestingly, these isolates can maintain virulence and often present advantageous phenotypic attributes. Altogether, these studies suggest that the *las* system might be dispensable in activating QS networks and strategic virulence gene expression in *P. aeruginosa*.

Presumably, the rest of the systems can take over the role of the central system and maintain bacterial pathogenicity, which is highly influenced by environmental pressure. This fact motivated Lee and co-workers to search the mechanisms by which the *las* system function could be compensated (Lee et al., 2013). With this purpose, the authors screened a transposon mutant library of *P. aeruginosa* PAO1 under *las*-independent conditions and found that a mutation in the *ambB* gene resulted in a decrease of pyocyanin and elastase production, thus establishing a connection with Pqs and Rhl systems. Subsequently, PQS was selected as an indicator of the activation of the aforementioned system and further studies showed that deletion of either *ambB*, *ambC*, *ambD*, or *ambE* resulted in its decreased production. On the other hand, overexpression of these genes in a *lasI* and *pqsA* double deletion mutant resulted in an increased production of a different metabolite, then identified as IQS. The addition of IQS to an *ambB* mutant recovered the production of C4-HSL and PQS in a dose-dependent manner and also of related virulence factors. With these results, the authors demonstrate the cell-to-cell signaling function of IQS and the connection with Pqs and Rhl systems. Moreover, the authors demonstrated that the Iqs signaling system was regulated by the *las* system under a rich medium and, on the other hand, it was independent in low phosphate conditions, where its regulation was mainly in charge of the two-component phosphate-dependent regulatory system PhoR-PhoB (Santos-Beneit, 2015).

## 3 Biosynthesis of IQS

*P. aeruginosa* produces the oxyvinylglycine-type antimetabolite L-2-amino-4-methoxy-trans-3-butenoic acid (AMB), which is a non-proteinogenic amino acid that irreversibly inhibits pyridoxal phosphate-dependent enzymes such as aminotransferases in bacteria, plants and animals (Berkowitz et al., 2006). Prior to the proposal of the Iqs as a QS system, it was reported that the cluster *ambABCDE* was responsible for the biosynthesis of AMB (Lee et al., 2010). The authors partially based their biosynthesis hypothesis on the inhibition of *Escherichia coli* K12 by the action of AMB. Thereby, they observed inhibition when the cluster *ambBCDE* was expressed. However, three years later Lee and co-workers demonstrated that the production of AMB and the inhibition of *E. Coli* K12 was controlled and prompted by IQS (Lee et al., 2013). As expected, they showed that a *P. aeruginosa ambB* mutant, theoretically unable to produce AMB, did not show an inhibition zone. Nonetheless upon the addition of IQS, the production of AMB was restored and so was the inhibition. Unless the inhibition was caused by other derived products, they proved that AMB was not produced by the *ambBCDE* cluster and that indeed its production was under the control of the Iqs signaling system. The authors also proved that deletion of either *ambB, ambC, ambD*, and *ambE* diminished the production of IQS, while deletion of *ambA* did not affect IQS production since it encodes for a LysE-type transporter protein (Lee et al., 2013; Wang et al., 2014). Moreover, the overexpression of the cluster *ambBCDE* in *P. aeruginosa* PAO1 increased the IQS yield in comparison with a wild-type strain PAO1 (Lee et al., 2013). Furthermore, a study that demonstrated the virulent effect of IQS against eukaryotic cells, published that mice infected with a *P. aeruginosa ambB* null mutant strain showed longer lives than mice infected with the same strain of *P. aeruginosa* plus the addition of exogenous IQS (Wang et al., 2019). These results were in favour of the IQS biosynthesis by the *ambBCDE* cluster. For all these reasons, since 2013 several articles and reviews have been published assuming IQS to be the product of the *ambBCDE* cluster (Wang et al., 2014; Lee and Zhang, 2015; Papenfort and Bassler, 2016; Chahtane et al., 2018; Wang et al., 2018; Zhang et al., 2018). In contrast, several studies have shown that the *ambABCDE* cluster is responsible for the biosynthesis of AMB and that IQS is synthesized through an alternative pathway.

**Figure 2:**
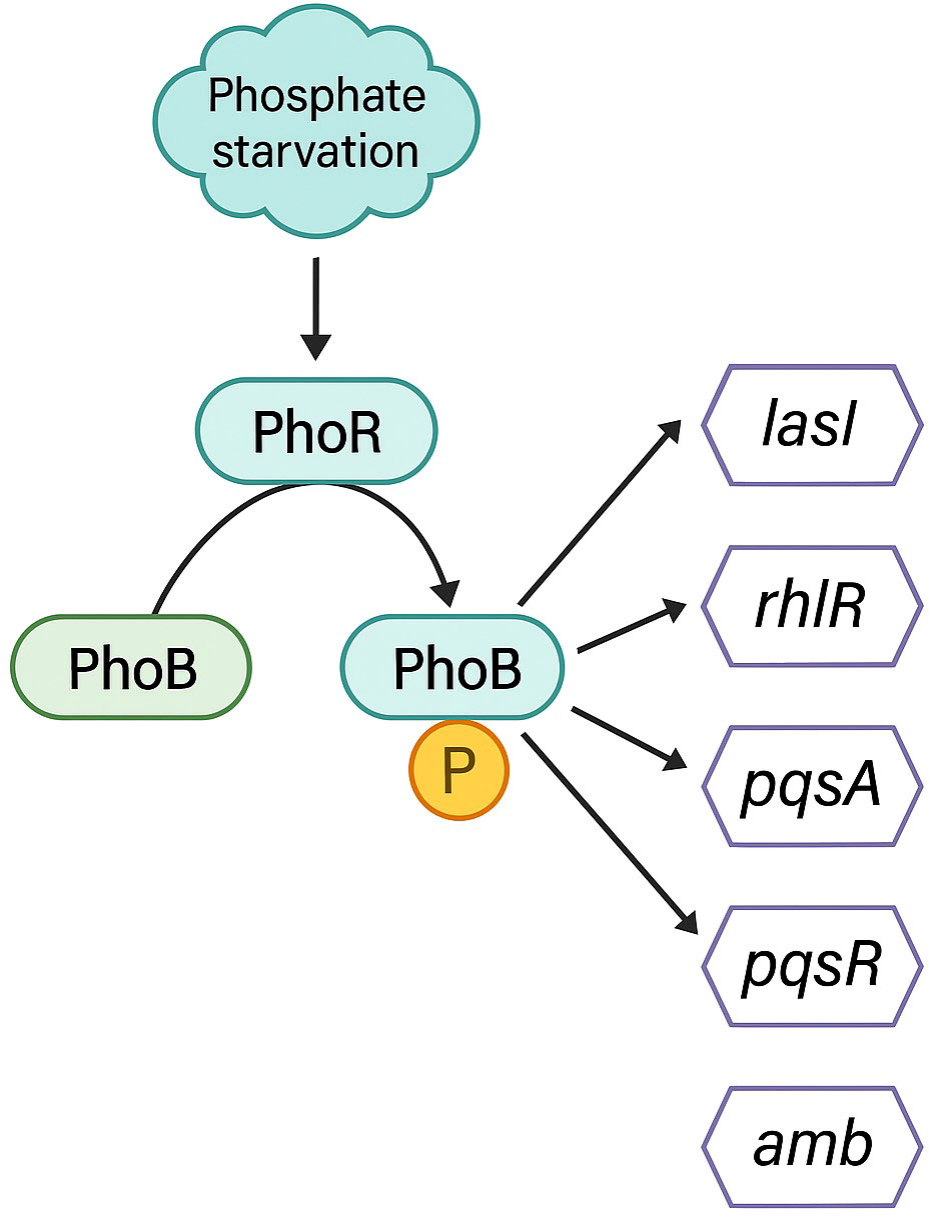
Schematic representation of the synthesis process of the antimetabolite AMB. Figure adapted from Murica et. al.

In favour of such second hypothesis is the work by Murcia and co-workers in 2015, considering that the *ambABCDE* cluster comprises two transcriptional units, one formed by *ambA* and the other by *ambBCDE*. According to them, the synthesis would be initiated by the activation of two L-Ala by AmbB, one loaded on the AmbB T domain and the other onto the AmbE T2 domain, both linked through a thioester bound to the enzyme and then, AmbE would load an L-Glu into its T1 domain. AmbB catalyzes the condensation between L-Ala and L-Glu to form the dipeptide L-Ala-LGlu at T1 of AmbE. The dipeptide would subsequently be condensed with the L-Ala residue loaded into the T2 domain of AmbE to form the tripeptide L-Ala-L-Glu-L-Ala. The central L-Glu would be modified by the monooxygenases AmbC and AmbD to form L-Ala-AMB-L-Ala; the order and the timing of the modifications are still unknown. It is believed that the two flanking L-Ala could act as protecting groups during the conversion of L-Glu into AMB and prevent toxicity of AMB inside the bacteria. It was also assumed that AmbA, a LysE-type transporter protein, is in charge of releasing the antimetabolite. However, it is not known if it is released as AMB or as L-Ala-AMB-L-Ala, which would be unprotected once inside the cell acting as a toxin (Rojas Murcia et al., 2015) (See **Figure 2**).

**Figure 3:**
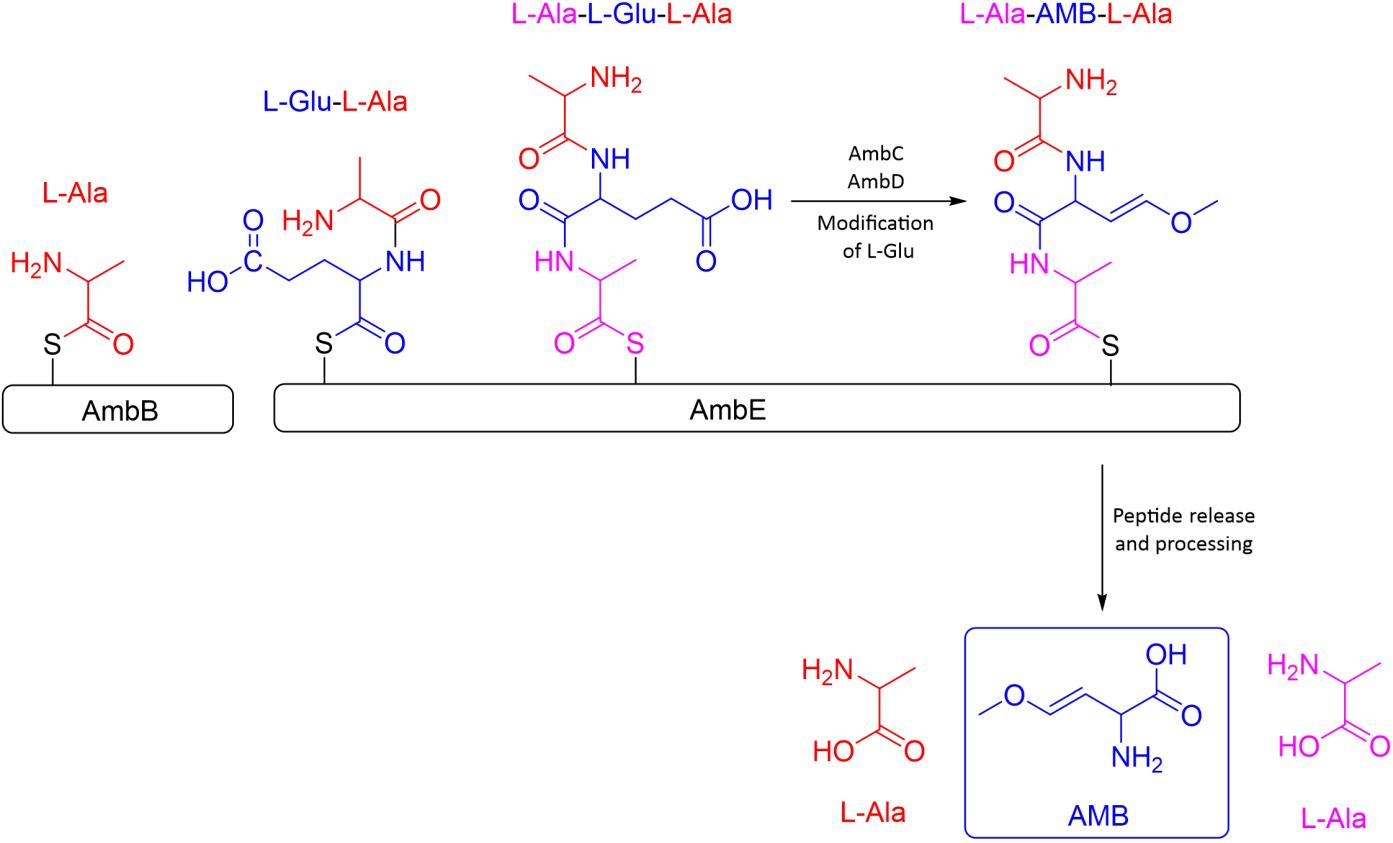
Biosynthesis pathway of PCH in *P. aeruginosa*. Dihydroaeruginoic acid is released during the assembly between PchE and PchF and can be transformed into IQS through simple dehydration.

The structural similarity between IQS and the siderophore PCH, suggests that IQS could be synthesized from PCH or from an intermediate pathway encoded by the *pch* gene. PCH is a siderophore produced by *P. aeruginosa* along with pyoverdine to internalize the available Fe^+3^, as it is an essential element for the bacteria’s survival. The production of PCH is regulated by the Fur system, which triggers the production of siderophores when it senses an iron limitation in the environment (Ochsner et al., 1995; Brandel et al., 2012). The cluster gene *pch* synthetizes PCH by the condensation of salicylic acid and L-Cys catalyzed by PchE, the L-Cys after a cyclization forms the thiazole ring. At this point of the synthesis, dihydroaeruginoic acid could be released, or another L-Cys could be added to form a second thiazole ring yielding PCH (Reimmann et al., 1998; Patel and Walsh, 2001; Gross and Loper, 2009) (see **Figure 3**). Although further studies are required, dihydroaeruginoic acid is probably transformed into IQS through simple dehydration (Ye et al., 2014).

**Figure 4:**
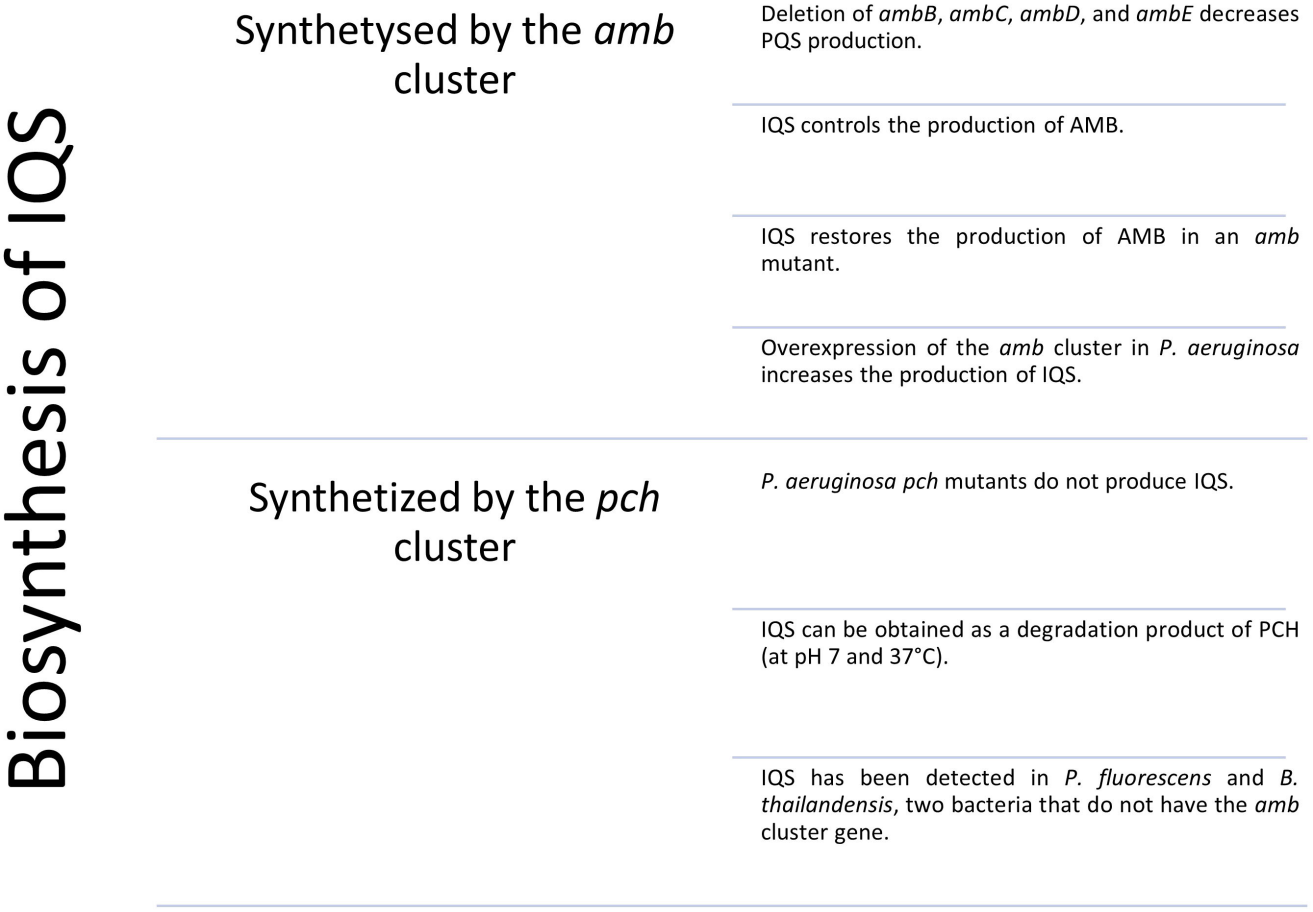
Schematic summary of the evidence for which it is believed that IQS is synthesized by the amb or pch gene cluster.

To prove that IQS is formed as a by-product in the synthesis of PCH, a strain of *P. aeruginosa* with the *pch* cluster suppressed was generated. An LC-MS measurement of the supernatant demonstrated that neither PCH nor IQS were produced by this mutant. Another proposed alternative is that IQS could be the result of the degradation of PCH since this siderophore decomposes into IQS at 30 °C in phosphate buffer (pH 7.0). Moreover, IQS has been found in the supernatant of *P. fluorescens* and *B. thailandensis* cultures, although both strains lack the *ambABCDE* gene (Trottmann et al., 2019). These reasons, point at the greater feasibility of the hypothesis that IQS was formed through the expression of the *pch* gene rather than from the *amb* cluster. However, there is also evidence that *ambBCDE* influences the production of IQS, therefore investigations are needed to clarify the biosynthesis of this molecule (See **Figure 4**).

## 4 Biological significance of IQS

### 4.1 Activation of QS systems under phosphate stress conditions

Phosphate is essential for all living organisms as a fundamental component of ATP, nucleotides, phospholipids, and other relevant biomolecules. Therefore, the ability to combat phosphate limitation conditions, by exploiting the available phosphate is critical for *P. aeruginosa* survival. A two-component regulatory system, PhoB and PhoR are in charge of sensing and signaling the phosphate deficiency. PhoR is an integral membrane signaling histidine kinase that senses environmental inorganic phosphate levels. Under conditions of phosphate limitation, PhoR activates the response regulator PhoB by phosphorylation. Once activated, PhoB regulates the expression of a set of genes by binding to a *pho box*, a specific DNA sequence in the promoter region (Lamarche et al., 2008; Faure et al., 2013). The *pho box* is typically present in genes that encode proteins important for phosphate assimilation or metabolism, such as PstS protein (Lamarche et al., 2008; Zaborina et al., 2008). Some evidences suggest a link between the Pho regulon and the expression of virulence factors, the promotion of swarming motility, and the regulation of biofilm formation (Jensen et al., 2006; Haddad et al., 2009; Blus-Kadosh et al., 2013).

Several authors have published that under phosphate stress conditions, a common environment during infections, *P. aeruginosa* produces a more virulent phenotype than under regular conditions (Long et al., 2008; Zaborin et al., 2009; Bains et al., 2012). Lee and co-workers reported that under normal culture conditions, Iqs was controlled by the Las system, but under deficiency of phosphate PhoB enhanced the production of IQS (Lee et al., 2013). A higher production of IQS would trigger the activation of Pqs and Rhl systems and consequently a boost in QS-related virulence factors, explaining the increase in the virulence of this phenotype. The activation of the Iqs system by PhoB instead of Las could explain the frequently detected *P. aeruginosa* clinical isolates with mutations in *lasIR*, but still maintaining the virulent phenotype, since Iqs can take over the functions of the central Las system. Moreover, it can also explain the reported *las*-independent activation of Pqs and Rhl systems (Jensen et al., 2006; Dekimpe and Deziel, 2009).

Very recently, a study proved that PhoB upregulates the expression of *lasI, rhlR, pqsA*, and *pqsR (* Meng et al., 2020). Surprisingly, the concentration of 3-oxo-C12-HSL did not increase. Further investigation showed that PhoB also enhanced the expression of the *pvdQ* gene, encoding an acyl-homoserine lactone acylase responsible for the degradation of 3-oxo-C12-HSL, explaining why increased *lasI* expression did not translate into an accumulation of 3-oxo-C12-HSL (Nadal Jimenez et al., 2010; Meng et al., 2020). The upregulation of genes corresponding to Rhl and Pqs QS systems under phosphate depletion conditions is in accordance with the reported increase of pyocyanin, rhamnolipids, elastase, PQS, and C4-HSL (Jensen et al., 2006; Haddad et al., 2009; Zaborin et al., 2009; Bains et al., 2012). The null mutation of *phoB* completely suppresses the virulence of *P. aeruginosa* and diminishes its swarming motility under phosphate limitation conditions (Zaborin et al., 2009; Bains et al., 2012).

**Figure 5:**
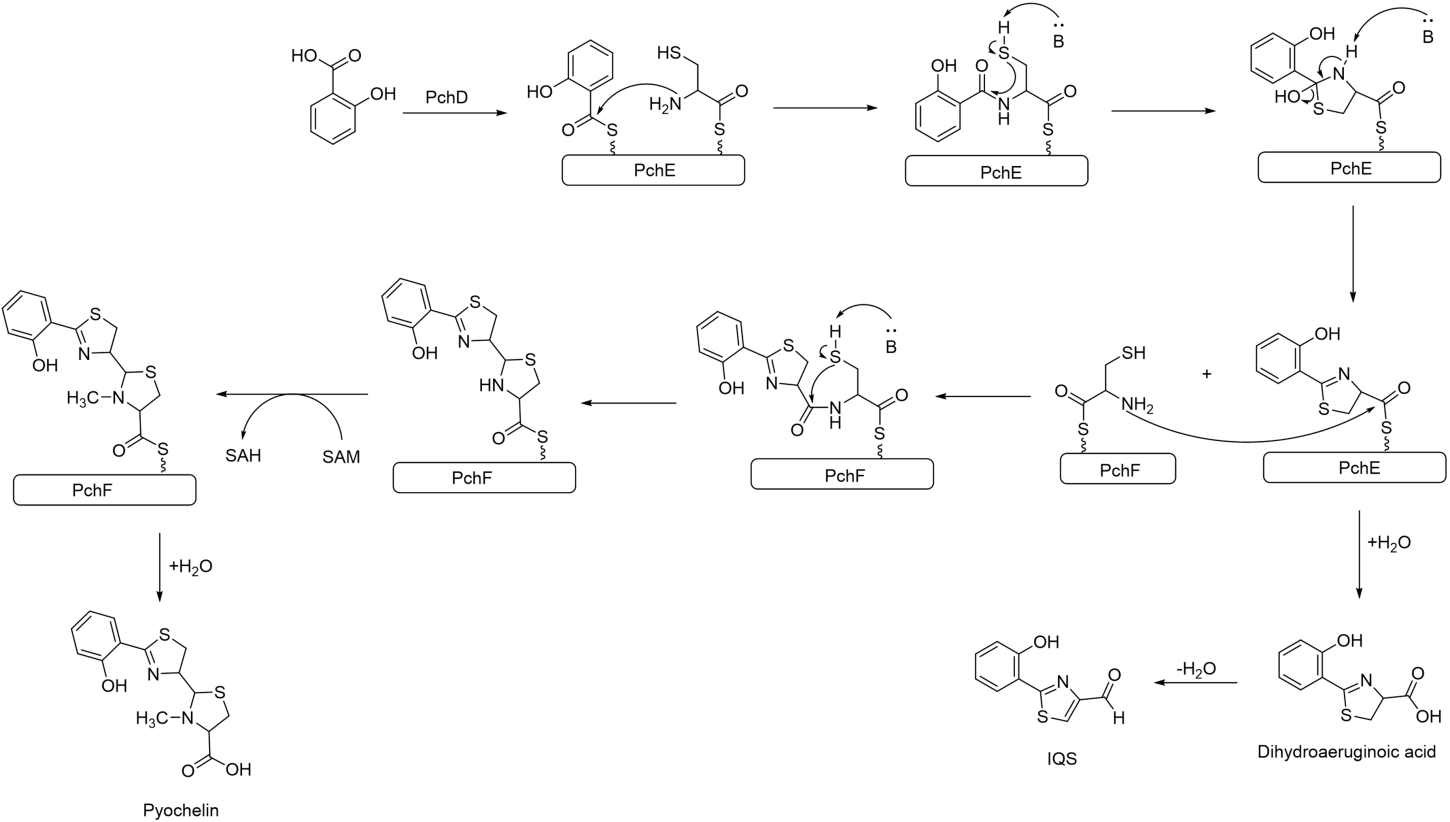
Schematic representation of the QS-related genes induced by the PhoB regulon under phosphate depletion.

The phosphate regulon PhoB has been demonstrated to directly activate the four QS systems in *P. aeruginosa*. For the *las* system, a *pho box* was found in the promoter of the *lasI* gene. The affinity of PhoB for the *lasI* gene was confirmed through an electrophoretic mobility shift assay (EMSA) (Meng et al., 2020). For the *rhl* system, in the promoter region of the *rhlR* gene, a putative PhoB binding site was found (Jensen et al., 2006). Using ChIP-seq analysis, *pqsA* and *pqsR* were found to have a *pho box* in their promoter regions (Bielecki et al., 2015). Eventually, as explained in section 2, the synthesis of IQS was also found to be enhanced by the phosphate regulon (Lee et al., 2013) (see **Figure 5**).

### 4.2 Influence of IQS on biofilm formation

The formation of biofilm is under the control of different genes and environmental factors (Yan and Wu, 2019). The QS controls part of the genes involved in either formation and morphology of biofilm (Davies et al., 1998; Wolska et al., 2016). For instance, it is broadly known that the production of rhamnolipids is regulated by the QS, and rhamnolipids are essential for the formation of channels inside the biofilm (Stoodley et al., 1994). Different authors have reported that the Pqs system is closely related to the formation of biofilm since a certain concentration of PQS is needed to maintain the biofilm matrix. In addition, Pqs is the only system that can be downregulated by another QS system. Thus, it could be responsible for the reversibility in the formation of biofilm (Yan and Wu, 2019). As described above, under phosphate-limiting conditions, Iqs takes over the control of Pqs and Rhl, being therefore responsible for biofilm formation (Dogan et al., 2019). On the other hand, in 2018 Li and co-workers reported IQS derivatives showing anti-biofilm activity resulting from the reaction of thiazole-4-carboxylic acid with different linear alcohols (R-OH) or amines (R-NH_2_). A compound named B-11 (3-chlorophenethyl thiazole-4-carboxylate) was observed to inhibit biofilm formation only under phosphate-limiting conditions. Increasing B-11 concentration significantly reduced the expression of *rhlA-gfp* and *pqsA-gfp*, but *lasB-gfp*. These observations pointed at the anti-biofilm activity through IQS pathways. B-11 was found to interact with the Iqs system, subsequently downregulating the expression of Pqs and Rhl and reducing both the formation of biofilms and the production of virulence factors (Li et al., 2018).

### 4.3 IQS as a virulence factor

It was recently published by Wang and co-workers that IQS inhibits cell growth and stimulates apoptosis of host cells. When IQS was added to human pulmonary epithelial cells, the growth was inhibited by 70-85%. The apoptosis of the cells was induced by triggering its DNA damage response but without causing physical damage to the DNA. The study demonstrated that IQS reduces the expression level of POT1, which activates the downstream p53 in an ATM/ATR-dependent manner. Although further studies are required to understand the complexity of these mechanisms, it is clear that IQS acts as a bioactive molecule in eukaryotic systems (Wang et al., 2019). This is not the only mechanism of *P. aeruginosa* to induce the apoptosis of eukaryotic cells. In 2014 Broquet and co-workers reviewed the virulent factors produced by *P. aeruginosa* that induce the apoptosis of host cells. These toxins can be delivered through the type III secretion system directly into the cytosol of the host cell, such as ExoS, T, U, and Y that induce the caspase apoptosis pathway. Moreover, secreted virulent factors like pyocyanin, exotoxin A, ExlA, and the QS signaling molecule 3-oxo-C12-HSL have been shown to induce the apoptosis of different cell lines (Broquet and Asehnoune, 2015). The virulence of IQS provides more complexity to the host-pathogen interaction during infections. In this context, it has been found that IQS derivatives, such as the previously mentioned B-11, reduced the production of rhamnolipid and pyocyanin under phosphate limitations (Li et al., 2018).

## 5 Detection of IQS

While *P. aeruginosa* AIs (PQS, HHQ, C4-HSL and 3-oxo-C12-HSL) and certain virulence factors (HQNO, pyocyanin) have been determined in bacteria cultures and clinical samples (sputum, plasma, and urine) (Barr et al., 2015; Montagut et al., 2020; Montagut et al., 2021; Rodriguez-Urretavizcaya et al., 2021; Montagut et al., 2022), pointing at the potential value of these molecules as biomarkers of infection, few analytical methodologies have been developed to detect IQS. To our knowledge, only one method for the quantification of IQS has been reported, although no data on its implementation for the measurement of complex biological matrices has been reported. The method is an electrochemical biosensor using boron-doped diamond (BDD) and glassy carbon (GC) electrodes. IQS quantification was carried out through simple cyclic voltammetry and amperometry. The reported detection limits are 12 and 86 nM for the BDD and the GC electrodes, respectively (Shang et al., 2014).

## 6 Summary and perspective

*Pseudomonas aeruginosa* is an opportunistic pathogen responsible for numerous nosocomial infections. It can colonize various environmental habitats and can survive and grow even under nutrient-poor and hostile conditions (Filiatrault et al., 2006; Frimmersdorf et al., 2010). The success of *P. aeruginosa*, as an opportunistic pathogen, is partly attributed to the bacterial population´s capacity to coordinate the genetic expression by using QS. It has been proved that QS contributes to changes in the clinical status of patients (Barr et al., 2015) and thus, it is of vital importance to fully understand its role, implication, and mechanism in bacterial pathogenicity. Over the last two decades, several articles about the Las, Rhl, and Pqs QS systems have been published, though there are still doubts in respect to the Iqs system which is considered by some researchers as the fourth QS system while others seem to have clear that is not. It has been considered a key system that explains the behavior of *P. aeruginosa* clinical isolates containing loss-of-function mutations in *lasI* and *lasR* genes (D’Argenio et al., 2007; Dekimpe and Deziel, 2009; Hoffman et al., 2009; Ciofu et al., 2010) and the reported las-independent activation of Rhl and Pqs systems under phosphate depletion (Jensen et al., 2006; Long et al., 2008).

Initially, Lee and co-workers reported that the genes responsible for the biosynthesis of IQS were the *amb* cluster (Lee et al., 2013). Further investigation showed that the *amb* cluster was responsible for synthesising the antimetabolite AMB (Rojas Murcia et al., 2015). Moreover, it was found that *P. protegens* and *Burkholderia thailandensis* were also able to produce IQS without having the *amb* cluster (Ye et al., 2014; Trottmann et al., 2019), which is against considering the *amb* cluster responsible for the biosynthesis of IQS. However, there must be an unknown connection between *amb* and IQS, to justify the results reported by Lee et. al.

The structural similarity between IQS and PCH suggests that IQS may be a by-product of PCH synthesis. The 2-(2-hydroxyphenyl)-thiazoline motif is commonly found in iron-chelating molecules or “siderophores” in pathogenic bacteria and are released in response to low-iron conditions in the host. IQS could, potentially, be related to bacterial siderophores, perhaps as a precursor or a degradation product. It was first identified as a reduction product of aeruginoic acid, itself produced by the incomplete biosynthesis of pyochelin (Budzikiewicz, 2004). Years later it was proven that PCH could decompose into IQS under mild conditions (30 °C and pH 7) or that IQS could be generated through dehydration of dihydroaeruginoic acid, a product released in the assembly between PchE and PchF in the synthesis of the siderophore (Ye et al., 2014; Trottmann et al., 2019).

In 2013, Lee and co-workers reported that under normal culture conditions, the production of IQS was induced by the Las system. However, under phosphate depletion, the Pho regulon controls the production of IQS. This study was performed considering that IQS was synthesized by the *amb* cluster, though now there are arguments against this assumption (Lee et al., 2013). Considering that IQS is synthesized by *pch* rather than *amb*, it is known that *pch* is activated under low iron conditions (Reimmann et al., 1998). Surprisingly, Zaborin and co-workers reported that the production of PCH was enhanced under phosphate depletion as well (Zaborin et al., 2009). Further investigation into the interconnectivity between the systems triggered under phosphate and iron stress in *P. aeruginosa* is needed to understand the mechanisms inducing the production of IQS. Under phosphate limiting conditions, regulon PhoB can directly induce the expression of *lasI*, *rhlR*, *pqsA*, *pqsR*, and the production of IQS (Jensen et al., 2006; Lee et al., 2013; Bielecki et al., 2015; Meng et al., 2020). The IQS signaling mechanism provides an explanation for the loss of Las QS regulation due to spontaneous nonsynonymous mutations as an adaptive trait for clinical isolates from chronic CF lung infections. Discovery of IQS unlocked a new paradigm for *P. aeruginosa* cell-cell communication in which inactivation of the Las QS system no longer disarms QS as a regulator for virulence but unveils a sophisticated chemical communication system that could integrate QS with environmental stress response to regulate the expression of downstream genes. However, Lee and co-workers reported that IQS can induce the expression of *rhlB*, *rhlR*, and *pqsA* under the same environmental conditions (Lee et al., 2013). This finding increases the complexity of the interconnectivity between QS and the systems triggered as a response to environmental stress. Much remains to be determined to understand the regulatory mechanisms and signaling network of the IQS system, for example, the identity of the IQS receptor and the role of the AmbCDBA product in IQS signaling. Although much progress has been made in understanding the chemical communication systems in *P. aeruginosa*, there are still numerous questions about the conditions or factors that trigger some of the signaling pathways and signaling intermediates.

Identification and characterization of bacterial chemical signaling systems are not only critical for the elucidation of general bacterial virulence regulatory mechanisms under laboratory conditions but also essential for understanding bacterial performance and behaviours under *in vivo* or natural environmental conditions. Opportune production and perception of these chemical signals allow the bacterial pathogen to evade host immune responses, invade, and disseminate in tissues by switching infection modes accordingly. The multiple chemicals signaling systems of *P. aeruginosa* QS highlight its high capacity to adapt to the environment, coordinating its social lifestyles and survival in host tissues through sophisticated and complicated genetic regulatory networks. Moreover, its usual subsistence in polymicrobial environments, determines that these signals also play a role in competition and cooperation within the community.

Identification of the key cell-cell signaling mechanism, including microbial ecologic interactions, opens new avenues for the development of new strategies to control bacterial infections targeting this QS system (Wu et al., 2020; Vashistha et al., 2023). Therapeutic targeting bacteria QS communication systems will require a deep knowledge of the hierarchical arrangement of signaling mechanisms and potential cross-talk that allow the pathogen to adapt and act effectively in a fluctuating chemical environment in host cells. As an example, we have shown here the activity of B-11, an IQS-related molecule inhibiting biofilm formation under phosphate-limiting conditions. According to the authors, increasing concentrations of B-11 significantly reduced the expression of *rhlA-gfp* and *pqsA-gfp*, but *lasB-gfp*, causing a reduction in the production of virulence factors such as rhamnolipid and pyocyanin under phosphate limitation. Other still unexplored strategies could involve quenching the IQS activity through different mechanisms including favoring its degradation of the inhibition of the biosynthetic pathway. However, multiple hurdles such as potential up regulation of related QS pathways triggering virulence or unintended immunomodulatory effects of the host, need to be deeple studied and evaluated on *in vitro* an *in vivo* experimental studies. There are still too many questions surrounding the role of IQS in pathogenicity, which should be investigated in more detail. Thus, it is not entirely clear the role and IQS functions in pathogenesis or regulating the QS pathways. Additionally, the receptor and the gene cluster responsible for its biosynthesis have not been fully identified. Without a clear understanding of these fundamental aspects, designing drugs specifically targeting the IQS system is still far. Despite these observations, AMR is one of the top global public health threats and there is not sufficient investment on developing new antimicrobial therapies according to the last 2023 WHO report as big pharma continues to walk away from investment in new antibiotics (WHO, 2024). There is a clear need to investigate and develop innovative pathogen specific treatments that minimize development of AMR. In this context QS, but also generating deep knowledge on the role of IQS in pathogenesis should be envisaged as a noteworthy possibility to develop new therapeutic strategies. Validation studies should be performed in real polymicrobial environments considering the complexity of these communication networks and considering also interactions with the host.
