# Correction: Analysing the integrated quorum sensing (iqs) system and its potential role in *Pseudomonas aeruginosa* pathogenesis

**DOI:** 10.3389/fcimb.2025.1634997

**Published:** 2025-06-11

**Authors:** Juan Raya, Enrique-J. Montagut, M.-Pilar Marco

**Affiliations:** ^1^ Department of Chemical and Biomolecular Nanotechnology, Nanobiotechnology for Diagnostics (Nb4D), Institute for Advanced Chemistry of Catalonia (IQAC) of the Spanish Council for Scientific Research (CSIC), Barcelona, Spain; ^2^ CIBER de Bioingeniería, Biomateriales y Nanomedicina (CIBER-BBN), Barcelona, Spain

**Keywords:** *P. aeruginosa*, quorum sensing, IQS, autoinducers, aeruginaldehyde

In the published article, there was an error in the article title. Instead of “Analysing the *integrated quorum sensing* system its potential role in *Pseudomonas aeruginosa* pathogenesis”, it should be “Analysing the integrated quorum sensing (iqs) system and its potential role in *Pseudomonas aeruginosa* pathogenesis”.

The authors apologize for this error and state that this does not change the scientific conclusions of the article in any way. The original article has been updated.

